# Malignant Catatonia Warrants Early Psychiatric-Critical Care Collaborative Management: Two Cases and Literature Review

**DOI:** 10.1155/2017/1951965

**Published:** 2017-01-30

**Authors:** Julia Park, Josh Tan, Sylvia Krzeminski, Maryam Hazeghazam, Meghana Bandlamuri, Richard W. Carlson

**Affiliations:** ^1^Department of Medicine, Maricopa Medical Center, Phoenix, AZ, USA; ^2^Department of Psychiatry, Maricopa Medical Center, Phoenix, AZ, USA; ^3^College of Medicine, University of Arizona, Phoenix, AZ, USA; ^4^College of Medicine, Mayo Clinic, Scottsdale, AZ, USA

## Abstract

Malignant catatonia (MC) is a life-threatening manifestation which can occur in the setting of an underlying neuropsychiatric syndrome or general medical illness and shares clinical and pathophysiological features and medical comorbidities with the Neuroleptic Malignant Syndrome (NMS). The subsequent diagnosis and definitive therapy of MC are typically delayed, which increases morbidity and mortality. We present two cases of MC and review recent literature of MC and NMS, illustrating factors which delay diagnosis and management. When clinical features suggest MC or NMS, we propose early critical care consultation and stabilization with collaborative psychiatric management.

## 1. Introduction

MC, previously termed “lethal catatonia,” is the most severe manifestation within the spectrum of catatonic syndromes. Catatonia is defined as immobility, rigidity, mutism, posturing, excessive motor activity, stupor, negativism, staring, and echolalia. MC represents a life-threatening manifestation that can develop in the context of a neuropsychiatric syndrome or general medical illness and includes behavioral changes, movement disturbances, and autonomic dysregulation [1–5].

Schizophrenia is the most commonly cited inciting condition, although major depression and various toxic-metabolic etiologies have also been implicated. Typical symptoms of MC include catalepsy, stupor, mutism, waxy flexibility, negativism, posturing, autonomic dysfunction, rigidity, fever, and muscle injury [4, 6–10].

Although the potential lethal consequences of MC are well known, diagnosis is often difficult and typically delayed. Due to the fact that both MC and NMS have similar biochemical and neuropharmacologic disturbances, similar clinical features can be seen in both disorders. Current concepts suggest that NMS and malignant catatonia represent a spectrum with biochemical and neuropharmacologic disturbances that involve disturbances of dopamine and GABAergic receptors [11, 12].

The predominant pathophysiology of NMS is central dopamine receptor blockade in the hypothalamus that results in autonomic dysregulation [13]. However, as many patients with either MC or NMS respond to benzodiazepines, it is thought that both share a single biochemical pathway of reduced GABA_A_ inhibition of the frontal corticostriatal tracts [6, 12].

Standard treatment of MC includes initiation of benzodiazepines and electroconvulsive therapy (ECT). Prior to this approach, the mortality of MC typically exceeded 50%. Recent management strategies which include combined ECT and benzodiazepines result in decreases in morbidity and mortality [14]. Dąbrowski et al. reported full recovery to baseline in up to 80% of patients with this approach [14, 15]. However delay of treatment continues to remain a concerning issue, as recent studies reveal treatment delays are on average 60 days prior to initiation of treatment with benzodiazepines or ECT [16].

We describe two patients with MC that illustrate the problems of misdiagnosis, delayed identifications, and management of critical cardiopulmonary and metabolic disturbances which lead to further delays of definitive therapy. We review clinical features and current management of MC and NMS and propose guidelines for prompt and ongoing collaborative management between psychiatry and critical care services when MC is suspected.

### 1.1. Case  1

A 55-year-old male with a history of schizophrenia who was noncompliant of medications presented to the hospital with catatonic symptoms and autonomic instability. During the hospital stay, the patient exhibited worsening catatonia, mutism, anorexia, leukocytosis, and autonomic instability. He was treated with rehydration, electrolyte replacement, antibiotics for aspiration pneumonia, and nasoenteral tube feeding. The clinical picture was initially thought to be most consistent with catatonic schizophrenia and the patient was transferred to a psychiatric facility for ECT after fourteen days of medical therapy. During ECT without protection of airway, the patient developed a cardiac arrest with pulseless ventricular tachycardia that was related to dehydration, pneumonia, and sepsis. He was resuscitated, intubated, and admitted to the ICU. The patient had additional complications which included Methicillin Sensitive* Staphylococcus aureus* (MSSA) pneumonia, ischemic colitis, and Vancomycin Resistant Enterococcus (VRE) Urinary Tract Infection (UTI). From the initial presentation to the facility after the cardiac arrest, the diagnosis was delayed by 15 days. Benzodiazepines were initiated at the time of diagnosis and ECT was initiated after medical stabilization that required a total of 26 days from initial presentation. The patient slowly showed signs of clinical improvement in mental status. He was discharged to an extended care inpatient psychiatric facility after nearly 16 months of inpatient psychiatric care.

### 1.2. Case  2

A 20-year-old male was admitted to inpatient psychiatric care due to acute psychosis. He developed autonomic instability, catatonia, mutism, waxy flexibility, and agitation. He was transferred to a medicine service for treatment of suspected MC. After a poor response to benzodiazepines, institution of ECT was delayed from the time of diagnosis by seventeen days. The delay in treatment was primarily related to exclusion of alternative diagnoses. Critical care was consulted 7 days after admission for MC. The patient was ultimately given a total of 12 sessions of ECT, which resulted in gradual improvement after each dose. During the hospital stay, the patient developed multiple complications including dehydration, urinary tract infection due to* Escherichia coli*, and aspiration pneumonia. The patient's mental status improved. He was discharged home after 4 months of inpatient psychiatric care.

## 2. Review and Discussion

The cases presented herein illustrate that MC is an emergent and life-threatening illness that is accompanied by multiorgan dysfunction, including cardiopulmonary and metabolic defects requiring concurrent ICU and psychiatric care [1]. Both patients presented in this article suffered severe complications, including cardiopulmonary crisis, infections, dehydration, and prolonged hospital stay. We propose that suspicion of MC should prompt collaborative psychiatric and critical care management with early use of ECT if there is inadequate response to benzodiazepines therapy [7, 17].

We agree with Tuerlings et al. that the high mortality associated with MC is related in part to the failure of rapid and efficient exclusion of alternative diagnoses [18, 19]. The authors reported that delay to first and second treatment was 15 and 60 days, respectively [18]. A decrease in this delay should be a major goal as treatment delay and longer duration of symptoms without treatment have been associated with poorer clinical response [20]. We propose that organic causes be promptly narrowed by psychiatric history, medications changes, overdoses, metabolic derangements, and central nervous system infections. Any suspicion of MC should prompt admission to a critical care unit and stabilization as the diagnostic process proceeds. Currently, no biomarkers exist for MC to assist in diagnosis, although there appears to be an association of elevated d-dimer in the disease [21]. Use of this marker may be helpful in expediting detection of this disease. We further postulate that favorable outcomes may be achieved by collaborative management between critical care and psychiatry, with earlier detection and exclusion of alternative diagnoses, and prompt management of complications associated with MC.

Similarities exist between NMS and MC, thus confounding the picture of a potentially life-threatening disease. Therefore, a comparison of the differences and similarities between the two syndromes is warranted [7, 22–26]. Typically, MC is predominantly manifested by bizarre behavior and mutism, posturing, and catalepsy and with psychiatric disturbances. In contrast, NMS is classically linked to exposure of a neuroleptic agent or atypical antipsychotic, with prominent features of rigidity, autonomic dysfunction, fever, and stupor [4, 11, 26]. Both MC and NMS may lead to muscle injury, aspiration, and metabolic disturbance due to hyperthermia and altered mental status. However, prodromes of MC have psychiatric undertones of psychosis, agitation, stupor, mutism, or anxiety while NMS would present with acute onset of autonomic instability and extrapyramidal side effects after antipsychotic exposure [4, 11, 26]. Therefore, if history excludes exposure to an antipsychotic, the diagnosis of MC would be apparent [3, 4, 22].

Unfortunately, this defining characteristic may be blurred for providers, as the majority of patients with MC have a psychiatric history and therefore have been treated with antipsychotics.

In many instances, the deduction to the diagnosis of NMS or MC requires several days, during which time patients may already develop medical comorbidities. One of the largest series that reviewed the clinical course of MC was reported by Tuerlings et al. (2010). The mean lag time from first catatonic symptoms to first treatment with benzodiazepines or ECT was 15 days on average. The time lag between the first and second treatment was approximately 27 days. Reported overall improvement with treatment was 76% and complete remission was 58%. Mortality was 9%. Virtually, it is impossible to differentiate NMS from MC on clinical presentation and course. Importantly, both conditions require the same treatment consisting of withdrawal of antipsychotics and initiation of benzodiazepines and ECT as first-line treatments [18].

Whether the diagnosis is MC or NMS, both of the conditions have life-threatening consequences and therefore need ICU and psychiatric collaboration. Alternative diagnoses must be swiftly excluded and benzodiazepines must be utilized within 24 hours as first-line treatment for MC and NMS [18]. Additionally, ECT has been theorized as effective second- if not first-line treatment. ECT should be supplemented after or additionally with benzodiazepines [27–29]. Benzodiazepines are theorized to improve MC and NMS by increasing GABA_A_ activity. Likewise, the use of ECT may involve GABA activity by inducing a neural storm with increased GABA transmission and clinical improvement [7, 27].

MC typically has a complicated course that involves multiple medical comorbid conditions such as dehydration, aspiration pneumonia, electrolyte disturbances, cardiopulmonary instability, and thromboembolic phenomena.  Table 1 lists symptoms and clinical features and associated complications. Psychiatric care including the use of benzodiazepines and ECT is important, but identifying the appropriate level of medical support is crucial [5, 6, 19]. The psychiatric management includes sedative care and ECT treatment for up to 7 days. We suggest that critical care consultation and protection of airway may facilitate safer ECT by close monitoring and prompt care for life-threatening arrhythmias, airway protection, aspiration, hemodynamic instability, ischemia, and fluid and electrolyte balance [17, 30, 31].

## 3. Conclusion

The previous cases and review describe MC from diagnosis to management and complications. Delays or subtherapeutic treatment with ineffective and less aggressive methods increase morbidity and mortality [16].

No standardized treatment protocols exist for complications of MC, thus causing a delay and hindrance to proper treatment. Prompt identification and institution of life-saving treatments of ECT and pharmacologic therapy with benzodiazepines can be achieved by early critical care consultation for appropriate level of care that is equipped for attention to fluid-electrolyte balance, cardiopulmonary stabilization, and thermoregulation.

## Figures and Tables

**Table 1:** Complications associated with patient's clinical features.

Clinical features	Potential complications
Autonomic instability	Hypotension, hypertension, arrhythmias, myocardial ischemia, rhabdomyolysis, hemodynamic instability

Decreased movement	Thromboembolic disease, pressure ulcers, rhabdomyolysis

Decreased oral intake	Dehydration, hypovolemia, electrolyte derangements, inability to take oral medications

Catalepsy	Decreased airway clearance, aspiration pneumonia, hypoxemia, pneumonia
